# Assessment of human diploid genome assembly with 10x Linked-Reads data

**DOI:** 10.1093/gigascience/giz141

**Published:** 2019-11-26

**Authors:** Lu Zhang, Xin Zhou, Ziming Weng, Arend Sidow

**Affiliations:** 1 Department of Computer Science, Hong Kong Baptist University; 2 Department of Pathology, 300 Pasteur Dr, Stanford University, Stanford, CA 94305 USA; 3 Department of Computer Science, Stanford University, Stanford, CA 94305 USA; 4 Department of Genetics, 300 Pasteur Dr, Stanford University, Stanford, CA 94305 USA

**Keywords:** 10x Linked-Read sequencing, *de novo* assembly, diploid human genome, library preparation

## Abstract

**Background:**

Producing cost-effective haplotype-resolved personal genomes remains challenging. 10x Linked-Read sequencing, with its high base quality and long-range information, has been demonstrated to facilitate *de novo* assembly of human genomes and variant detection. In this study, we investigate in depth how the parameter space of 10x library preparation and sequencing affects assembly quality, on the basis of both simulated and real libraries.

**Results:**

We prepared and sequenced eight 10x libraries with a diverse set of parameters from standard cell lines NA12878 and NA24385 and performed whole-genome assembly on the data. We also developed the simulator LRTK-SIM to follow the workflow of 10x data generation and produce realistic simulated Linked-Read data sets. We found that assembly quality could be improved by increasing the total sequencing coverage (*C*) and keeping physical coverage of DNA fragments (*C_F_*) or read coverage per fragment (*C_R_*) within broad ranges. The optimal physical coverage was between 332× and 823× and assembly quality worsened if it increased to >1,000× for a given *C*. Long DNA fragments could significantly extend phase blocks but decreased contig contiguity. The optimal length-weighted fragment length (*W*${\mu _{FL}}$) was ∼50–150 kb. When broadly optimal parameters were used for library preparation and sequencing, ∼80% of the genome was assembled in a diploid state.

**Conclusions:**

The Linked-Read libraries we generated and the parameter space we identified provide theoretical considerations and practical guidelines for personal genome assemblies based on 10x Linked-Read sequencing.

## Background

The human genome holds the key for understanding the genetic basis of human evolution, hereditary illnesses, and many phenotypes. Whole-genome reconstruction and variant discovery, accomplished by analysis of data from whole-genome sequencing experiments, are foundational for the study of human genomic variation and analysis of genotype-phenotype relationships. Over the past decades, cost-effective whole-genome sequencing has been revolutionized by short-fragment approaches, the most widespread of which have been the consistently improving generations of the original Solexa technology [1, 2], now referred to as Illumina sequencing. Illumina's strengths and weaknesses are inherent in the sample preparation and sequencing chemistry. Illumina generates short paired reads (2 × 150 bp for the highest-throughput platforms) from short fragments (usually 400–500 bp [3]. Because many clonally amplified molecules generate a robust signal during the sequencing reaction, Illumina's average per-base error rates are very low.

The lack of long-range contiguity between end-sequenced short fragments limits their application for reconstructing personal genomes. Long-range contiguity is important for phasing variants and dealing with genomic complex regions. For haplotyping, variants can be phased by population-based methods [4, 5] or family-based recombination inference [6, 7]. However, such approaches are only feasible for common variants in single individuals or when a trio or larger pedigree is sequenced. Furthermore, highly polymorphic regions such as the human leukocyte antigen (HLA) in which the reference sequence does not adequately capture the diversity segregating in the population are refractory to mapping-based approaches and require *de novo* assembly to reconstruct [8]. Short-read/short-fragment data are challenged by interspersed repetitive sequences from mobile elements and by segmental duplications, and only support highly fragmented genome reconstruction [9, 10].

In principle, many of these challenges can be overcome by long-read/long-fragment sequencing [11, 12]. Assembly of Pacific Biosciences (PacBio) or Oxford Nanopore (ONT) data can yield impressive contiguity of contigs and scaffolds. In 1 study [13], scaffold N50 reached 31.1 Mb by hierarchically integrating PacBio long reads and BioNano for a hybrid assembly, which also uncovered novel tandem repeats and replicated the structural variants (SVs) that were newly included in the updated hg38 human reference sequence. Another study [14] produced human genome assemblies with ONT data, in which a contig N50 ∼3 Mb was achieved, and long contigs covered all class I HLA regions. A recent whole-genome assembly of NA24385 [15] with high-quality PacBio Circular Consensus Sequencing (CCS) reads generated contigs with an N50 of 15 Mb. However, long-fragment sequencing is hindered by extremely high cost (in the case of PacBio CCS) or low base quality (in the case of single-pass reads of either technology), hampering its usefulness for personal genome assembly.

Hierarchical assembly pipelines in which multiple data types are used is another approach for genome assembly [16]. For example, in the reconstruction of an Asian personal genome, fosmid clone pools and Illumina data were merged, but because fosmid libraries are highly labor intensive to generate and sequence, this approach is not generalizable to personal genomes. The “long fragment read” (LFR) approach [17], where a long fragment is sequenced at high depth via single-molecule fragmented amplification, reported promising personal genome assembly and variant phasing by attaching a barcode to the short reads derived from the same long fragment. However, because LFR is implemented in a 384-well plate, many long fragments would be labeled by the same barcodes, making it difficult for binning short reads, and the great sequencing depth required rendered LFR not cost-effective.

An alternative approach is offered by the 10x Genomics Chromium system, which distributes the DNA preparation into millions of partitions where partition-specific barcode sequences are attached to short amplification products that are templated off the input fragments. Because of the limited reaction efficiency in each partition, the sequencing depth for each fragment is too shallow to reconstruct the original long fragment, distinguishing this approach from LFR [18]. However, to compensate for the low read coverage of each fragment, each genomic region is covered by hundreds of DNA fragments, giving overall sequence coverage that is in a range comparable to standard Illumina short-fragment sequencing while providing very high physical coverage. Novel computational approaches leveraging the special characteristics of 10x Genomics data have already generated significant advances in power and accuracy of haplotyping [19, 20], cancer genome reconstruction [21, 22], metagenomic assemblies [23], and *de novo* assembly of human and other genomes [24–26], compared to standard Illumina short-fragment sequencing. While the uniformity of sequence coverage is not as good as with PCR-free Illumina libraries, 10x Linked-Read sequencing is a promising technology that combines low per-base error and good small-variant discovery with long-range information for much improved SV detection in mapping-based approaches [22, 27], and the possibility of long-range contiguity in *de novo* assembly [24, 26, 28].

**Figure 1:**
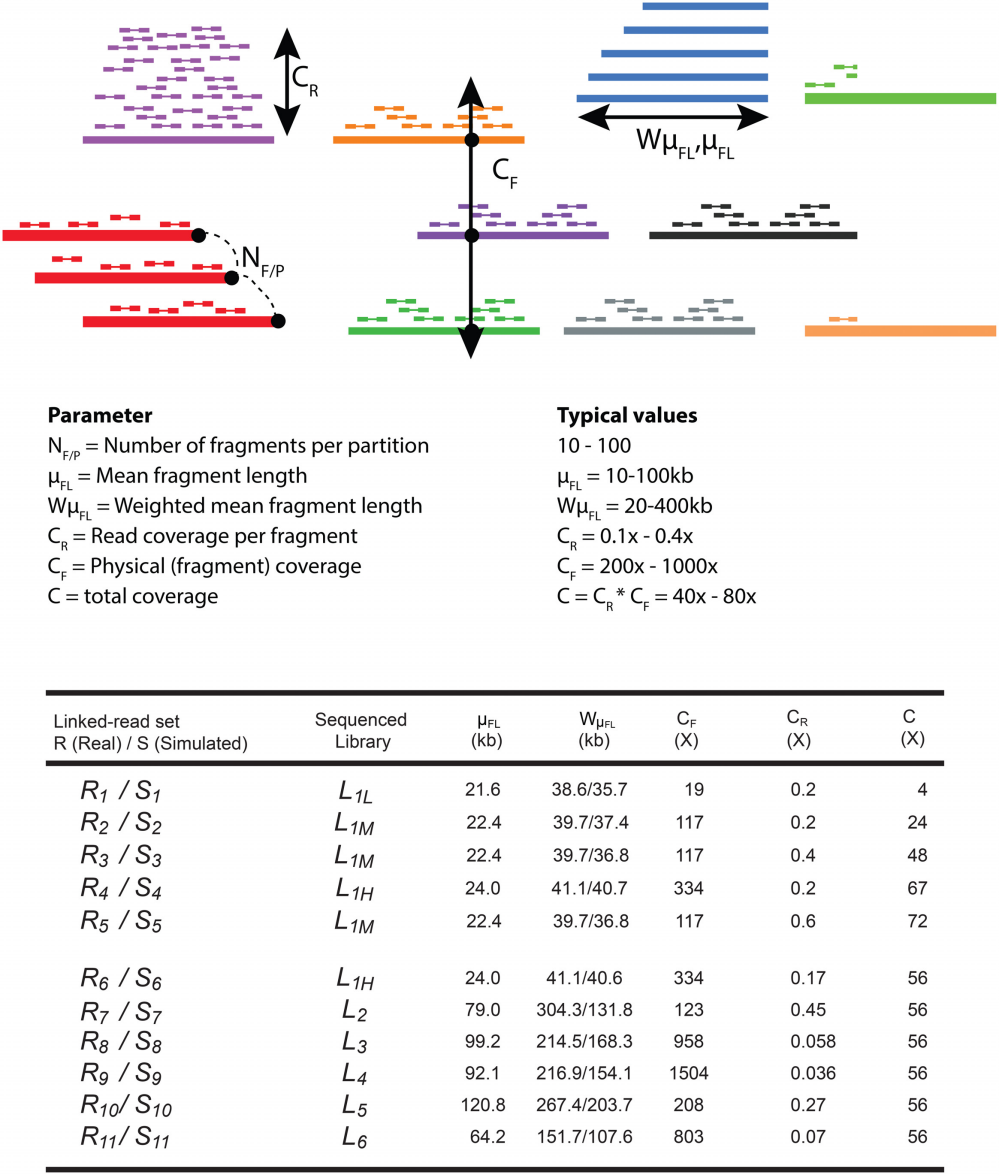
The Linked-Read sets prepared to evaluate the impact of *C_F_, C_R_*, ${\mu _{FL}}$, and *W*${\mu _{FL}}$ on human diploid assembly.

Practical advantages of the technology include the low DNA input mass requirement (1 ng per library, or ∼300 haploid human genome equivalents). Real input quantities can vary, along with other factors, to influence an interconnected array of parameters that are relevant to genome assembly and reconstruction. The parameters over which the experimenter has influence are (Fig. 1) as follows: (i) *C_R_*: average coverage of short reads per fragment; (ii) *C_F_*: average physical coverage of the genome by long DNA fragments; (iii) *N_F/P_*: number of fragments per partition; (iv) fragment length distribution, several parameters of which are used, specifically ${\mu _{FL}}$: mean unweighted DNA fragment length and *W*${\mu _{FL}}$: length-weighted mean of DNA fragment length. Note that several parameters depend on each other. For example, a greater amount of input DNA will increase *N_F/P_*; shorter fragments increase *N_F/P_* at the same DNA input amount compared to longer fragments; less input DNA will (within practical constraints) increase *C_R_* and decrease *C_F_*; and their absolute values are set by how much total sequence coverage is generated because *C_R_* × *C_F_* = *C*.

Our goal in this study was to experimentally explore the 10x parameter space and evaluate the quality of *de novo* diploid assembly as a function of the parameter values. For example, we set out to ask whether longer input fragments produce better assemblies, or what the effect of sequencing vs physical coverage is on contiguity of assembly. To constrain the parameter space, we first performed computer simulations with reasonably realistic synthetic data. The simulation results suggested certain parameter combinations that we then approximated in the generation of real, high-depth, sequence data on 2 human reference genome cell lines, NA12878 and NA24385. These simulated and real data sets were then used to produce *de novo* assemblies, with an emphasis on the performance of 10x's Supernova2 [24]. We finally assessed the quality of the assemblies using standard metrics of contiguity and accuracy, facilitated by the existence of a gold standard (in the case of simulations) and comparisons to the reference genome (in the case of real data).

## Methods

### Library preparation, physical parameters, and sequencing coverage

We made 6 DNA preparations that varied in fragment size distribution and amount of input DNA, 3 each from NA12878 (Coriell, Camden, NJ, USA; Cat. No. GM12878, RRID:CVCL_7526) and NA24385 (Coriell; Cat. No. GM24385, RRID:CVCL_1C78). From these, we prepared 8 libraries, 5 from NA12878 and 3 from NA24385 (Table S1). To generate libraries ${L_{1L}}$, ${\rm{\ }}{L_{1M}}$, and ${L_{1H}}$ (the subscripts *L, M*, and *H* represent low, medium, and high *C_F_*, respectively), genomic DNA was extracted from ∼1 million cultured NA12878 cells using the Gentra Puregene Blood Kit following manufacturer's instructions (Qiagen, Germantown, MD, USA; Cat. No 158 467). The emulsions (GEMs) were divided into 3 tubes with 5%, 20%, and 75% to generate libraries ${L_{1L}}$, ${L_{1M}}$, and ${L_{1H}}$, respectively (Figs S1–S3). For the other libraries, to generate longer DNA fragments (*W*${\mu _{FL}}$= 150 kb and longer, Figs S4–S8), a modified protocol was applied. Two-hundred thousand NA12878 or NA24385 cells of fresh culture were added to 1 mL cold 1× phosphate-buffered saline in a 1.5-mL tube and pelleted for 5 minutes at 300*g*. The cell pellets were completely resuspended in the residual supernatant by vortexing and then lysed by adding 200 µL Cell Lysis Solution and 1 µL of RNaseA Solution (Qiagen, Cat. No. 158 467), mixing by gentle inversion, and incubating at 37°C for 15–30 minutes. This cell lysis solution is used immediately as input for the 10x Genomics (Pleasanton, CA, USA) Chromium preparation (Chromium^TM^ Genome Library & Gel Bead Kit v2, PN-120 258; Chromium^TM^ i7 Multiplex Kit, PN-120 262). The fragment size of the input DNA can be controlled by gentle handling during lysis and DNA preparation for Chromium. The amount of input DNA (between 1.25 and 4 ng) was varied to achieve a wide range of physical coverage (*C_F_*).The Chromium Controller was operated and the GEM preparation was performed as instructed by the manufacturer. Individual libraries were then constructed by end repairing, A-tailing, adapter ligation, and PCR amplification. All libraries were sequenced with 3 lanes of paired-end 150-bp runs on the Illumina HiSeqX to obtain very high coverage (*C* = 94×–192×), although the 2 with the fewest gel beads (${L_{1L}}$ and ${L_{1M}}$) exhibited high PCR duplication rates because of the reduced complexity of the libraries (Table S1).

### Linked-Reads subsampling

The high sequencing coverage in the libraries allowed subsampling to facilitate the matching of parameters among the different libraries, for purposes of comparability; these subsampled Linked-Read sets are denoted ${R_{id}}$ (Fig. 1). We aligned the 10x Linked-Reads to human reference genome (hg38, GRCh38 Reference 2.1.0 from 10x website) followed by removing PCR duplication by barcode-aware analysis in Long Ranger [21]. Original input DNA fragments were inferred by collecting the read pairs with the same barcode that were aligned in proximity to each other. A fragment was terminated if the distance between 2 consecutive reads with the identical barcode >50 kb. Fragments were required to have ≥2 read pairs with the same barcode and a length of ≥2 kb. Partitions with <3 fragments were removed. We subsampled short reads for each fragment to satisfy the expected *C_R_*.

### Generating 10x simulated libraries by LRTK-SIM

To compare the observations from real data with a known truth set, we developed LRTK-SIM, a simulator that follows the workflow of the 10x Chromium system and generates synthetic Linked-Reads like those produced by an Illumina HiSeqX machine (Supplementary Information and Fig. S9). Based on the parameters commonly used by 10x Genomics Linked-Read sequencing and the characteristics of our libraries, LRTK-SIM generated simulated data sets from the human reference (hg38), explicitly modeling the 5 key steps in real data generation. Parameters in parentheses are from the standard 10x Genomics protocol: (i) shearing genomic DNA into long fragments (W${\mu _{FL}}$ from 50 to 100 kb); (ii) loading DNA to the 10x Chromium instrument (∼1.25 ng DNA); (iii) allocating DNA fragments into partitions to which are attached the unique barcodes (∼10 fragments per partition); (iv) generating short fragments; (v) generating Illumina paired-end short reads (800,000,000–1,200,000,000 reads). LRTK-SIM first generated a diploid reference genome as a template by duplicating the human reference genome (hg38) into 2 haplotypes and inserting single-nucleotide variants (SNVs) from high-confidence regions in (GIAB) of NA12878 [29]; for low-confidence regions we randomly simulated 1 SNV per 1 kb. The ratio was 2:1 for heterozygous and homozygous SNVs. From this diploid reference genome, LRTK-SIM generated long DNA fragments by randomly shearing each haplotype with multiple copies into pieces whose lengths were sampled from an exponential distribution with mean of ${\mu _{FL}}$. These fragments were then allocated to pseudo-partitions, and all the fragments within each partition were assigned the same barcode. The number of fragments for each partition was randomly picked from a Poisson distribution with mean of *N_F/P_*. Finally, paired-end short reads were generated according to *C_R_* and replaced the first 16 bp of the reads from the forward strand to the assigned barcodes followed by 7 Ns. More information about implementation can be found in the Supplementary Information. From that diploid genome, Linked-Read data sets were generated that varied in *C_R_, C_F_*, and ${\mu _{FL}}$ (*W*${\mu _{FL}}$) (Tables S2 and S3). Varying *N_F/P_* was only done for chromosome 19 because of the infeasibility of running Supernova2 on whole-genome assemblies with large *N_F/P_*; within practically reasonable values, *N_F/P_* does not seem to influence assembly quality (Fig. S10). In total, we generated 17 simulated Linked-Read data sets to explore the overall parameter space (Tables S2 and S3) and 11 to match the parameters of the aforementioned real libraries (Fig. 1).

LRTK-SIM provides more flexible simulation parameters than another method for simulating Linked-Read data, LRSIM [30]. It explicitly allows users to input *C_F_, C_R_, W*${\mu _{FL}}$, and ${\mu _{FL}}$, which have strong connections with library preparation and Illumina sequencing, whereas LRSIM only lets the user set the total number of reads. For example, *C_F_* is driven by input DNA amount, and ${\mu _{FL}}$ by DNA preparation and potential size selection. Also, LRSIM requires many third-party packages and software to be installed first, such as Inline::C perl library and DWGSIM [31]. By contrast, LRTK-SIM was written in Python and no third-party software is required to run it. LRTK-SIM can simulate multiple libraries with a variety of parameters simultaneously, and users can compare the performance of different parameters in 1 run.

### Human genome diploid assembly and evaluation

The scaffolds were generated by the “pseudohap2” output of Supernova2, which explicitly generated 2 haploid scaffolds, simultaneously. Contigs were generated by breaking the scaffolds if ≥10 consecutive N's appeared, per definition by Supernova2. For the simulations of human chromosome 19, we used the scaffolds from the “megabubbles” output. Contig and scaffold N50 and NA50 were used to evaluate assembly quality. Contigs longer than 500 bp were aligned to hg38 by Minimap2 [32]. We calculated contig NA50 on the basis of contig misassemblies reported by QUAST-LG [33]. For scaffolds (longer than 1 kb), we calculated the NA50 following Assemblathon 1’s procedure [34] (Supplementary Information, Evaluation of Diploid Assembly).

### Genomic variant calls from diploid assembly

We compare SNVs and SVs from the diploid regions of our assemblies with the ones from standard Illumina data and reference-based processing of our 10x data. The standard Illumina data were downloaded from GIAB [35] and analyzed with SvABA [36] to generate SV calls, and with BWA (BWA, RRID:SCR_010910) [37] and FreeBayes (FreeBayes, RRID:SCR_010761) [38] to generate SNV calls. Long ranger ([39]) was used to generate SNV and SV (only deletions) calls for 10x reference-based analysis. We note that *R*_9_ failed to be analyzed by Long Ranger owing to its extremely large *C_F_*. For SNVs, we compared the calls from 3 strategies using the benchmark of NA12878 [40] and NA24385 [41]. For SVs, we compared 3 Linked-Read sets (*R*_9_, *R*_10_, *R*_11_) from HG002 with the Tier 1 SV benchmark from GIAB [42] and used VaPoR [43] to validate our SV calls based on PacBio CCS reads from NA24385 [44]. We compared SNV and SV calls among the different approaches using vcfeval [45] and truvari [42], respectively.

## Results

### Performance of diploid assembly: influence of total coverage

**Figure 2:**
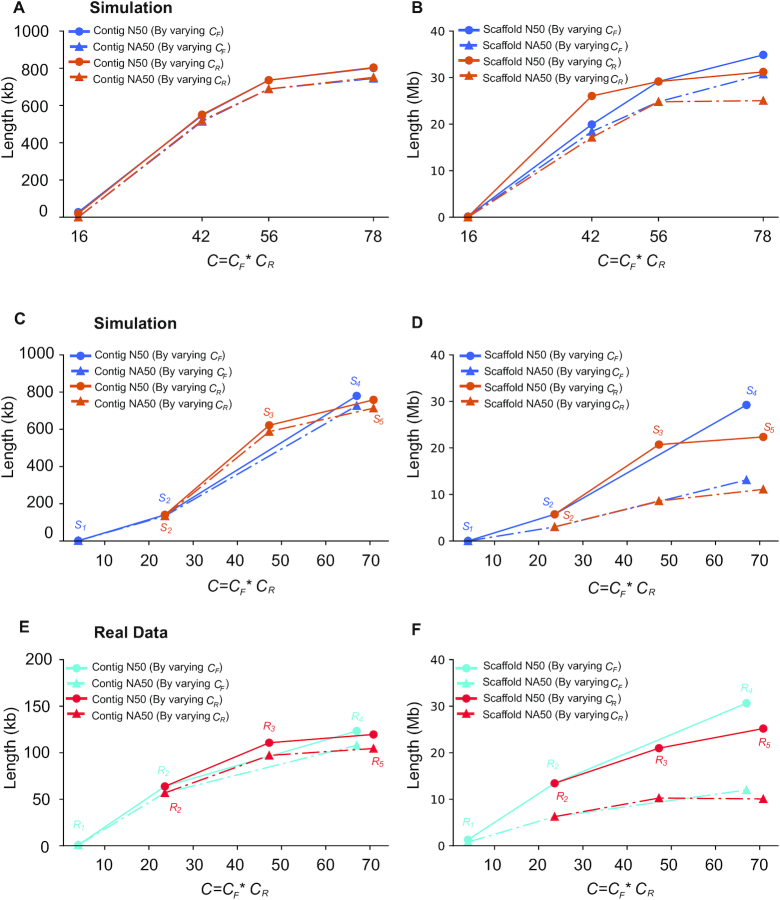
Contig and scaffold lengths (N50 and NA50) as a function of *C_F_* or *C_R_*. **A** and **B**: Simulated Linked-Reads with predefined parameters (Table S2); **C** and **D**: Simulated Linked-Reads with matched parameters of real Linked-Read data sets (Fig. 1); **E** and **F**: Real Linked-Read sets (Fig. 1).

Diploid assembly by Linked-Reads requires sufficient total read coverage (*C* = *C_R_* × *C_F_*) to generate long contigs and scaffolds. In this experiment, to explore the roles of both physical coverage (*C_F_*) and per-fragment read coverage (*C_R_*), we first generated 8 simulated libraries whose total coverage *C* ranged from 16× to 78×: 4 with *C_R_* fixed and increasing *C_F_* and 4 with fixed *C_F_* and increasing *C_R_* (Table S2). Contig and scaffold N50s increased along with increasing either *C_F_* or *C_R_* (Fig. 2A and B). To investigate whether the trend was also present in the real data sets, we analyzed 6 real libraries (3 by varying *C_F_* and 3 by varying *C_R_*; Fig. 1): as *C* increased, we varied *C_F_* and *C_R_* independently by fixing the other parameter. Contig and scaffold N50s also increased in these simulations (Fig. 2C and D) and real Linked-Read sets (Fig. 2E and F) as a function of total coverage *C*. Contig lengths did increase a little (621.4 to 758.1 kb for simulation; 110.7 to 119.6 kb for real data) when *C* was increased beyond 56×. Accuracy, which we define as the ratio between NA50 (N50 after breaking contigs or scaffolds at assembly errors) and N50 (Fig. 2C and E), changed 18% for simulation and 7% for real data (587.5 to 713.3 kb for simulation; 97.1 to 104.5 kb for real data). For scaffolds in the real data sets, when *C* increased from 48× (${R_3}$) to 67× (${R_4}$), both scaffold N50 and NA50 were significantly improved (N50: 13.4 to 30.6 Mb; NA50: 6.3 to 12.0 Mb), but the accuracy decreased slightly from 46.6% to 39.1%, which indicated that scaffold accuracy may be refractory to extremely high *C* (Fig. 2F). These results indicated that assembly length and accuracy were comparable over a broad range of *C_F_* and *C_R_* at constant *C*, which implied that assembly quality was mainly determined by *C*.

### Performance of diploid assembly: influence of fragment length and physical coverage

**Figure 3:**
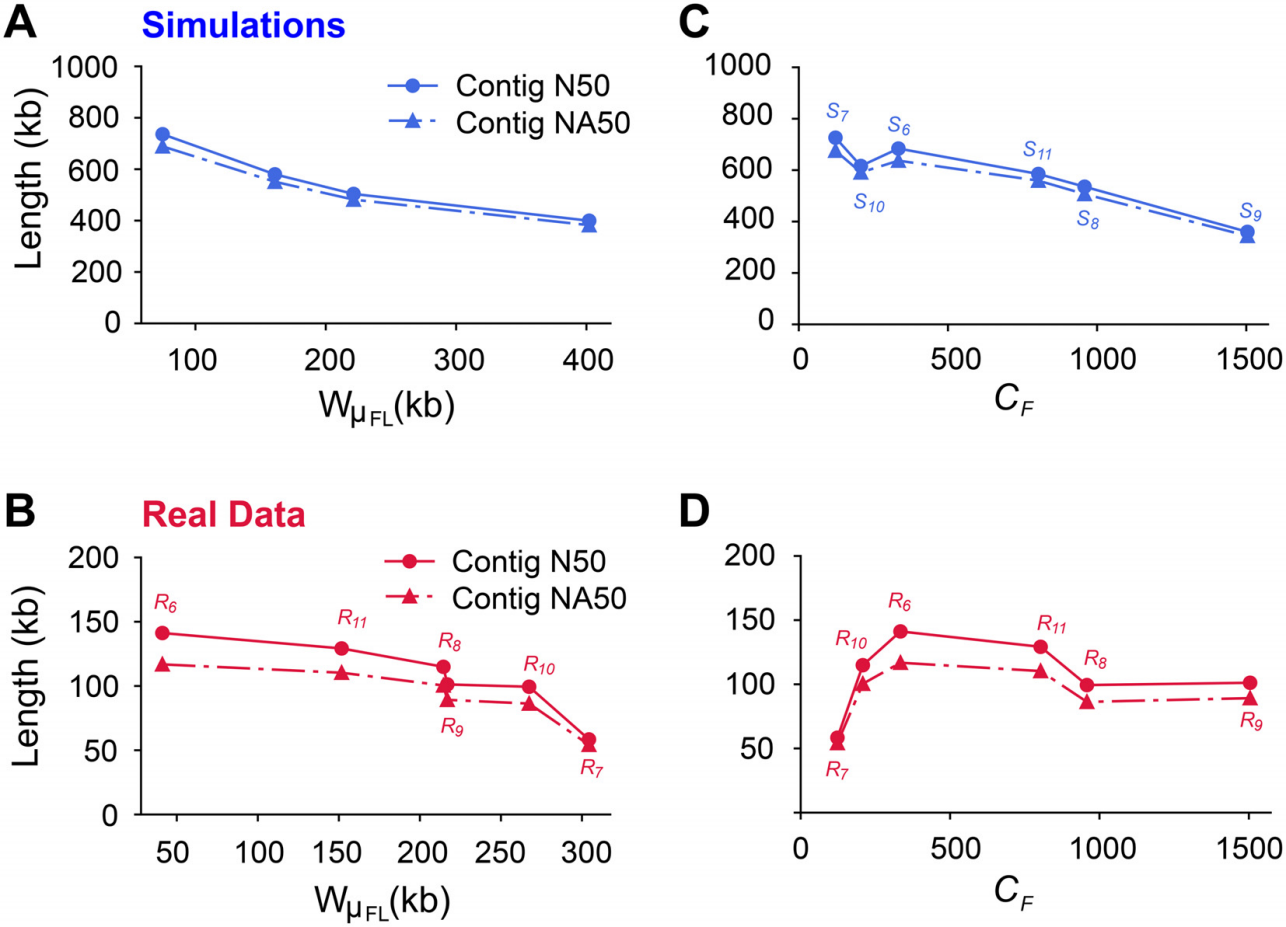
Contig qualities (N50 and NA50) as a function of fragment length $W{\mu _{FL}}$ or physical coverage *C_F_*, at *C* = 56×. **A** and **C**, results from simulations; **B** and **D**, results from real data.

To investigate whether input weighted fragment length (as measured by *W*${\mu _{FL}}$) influenced assembly quality, we generated 4 simulated libraries (Table S3) with fixed *C_F_* and *C_R_* and a range of fragment lengths (Fig. 3A). Contig length decreased with increasing fragment length, a trend that was also seen in 6 real libraries (Fig. 3B; *C* = 56×; ${R_6}$ to ${R_{11}}$ in Fig. 1). We then simulated another 6 libraries with the same parameters as the real ones to explore the effects of physical coverage at constant *C* = 56× (Fig. 3C). Contig lengths decreased as a function of increasing physical coverage, a trend that is somewhat less clear in real data possibly owing to confounding other parameters such as fragment length (Fig. 3D). The 2 Linked-Read sets with the worst contig qualities in NA12878 (${R_7}$) and NA24385 (${R_{10}}$) also showed a significant increase of the number of breakpoints (Table S4).

### Performance of diploid assembly: nature of the source genome

**Figure 4:**
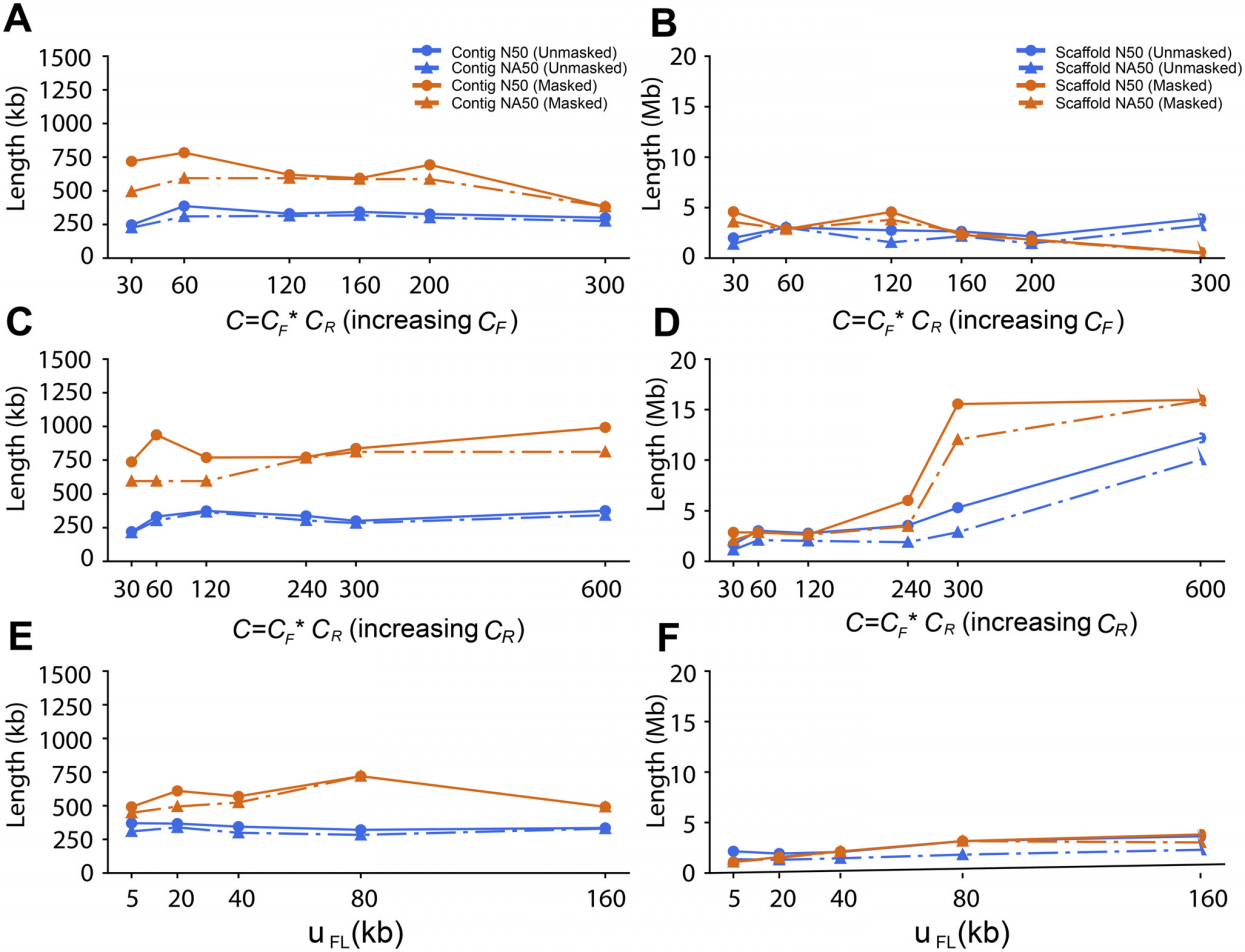
Comparison of contig and scaffold lengths from 10x data with masked and unmasked repetitive sequences by changing *C_F_, C_R_*, and ${\mu _{FL}}$. *C_R_* was fixed to 0.2× in **A** and **B**; *C_F_* was fixed to 300× in **C** and **D**; *C_R_* was fixed to 0.2× and *C_F_* was fixed to 300× in **E** and **F**.

Assembly errors may occur because of heterozygosity, repetitive sequences, or sequencing error. To illuminate possible sources of assembly error, we performed simulations by generating 10x-like Linked-Reads as above from human chromosome 19, and then quantified assembly error against these synthetic gold standards. Removal of interspersed repeat sequences from the source genome resulted in better contigs with no loss of accuracy in experiments by varying *C_F_, C_R_*, and ${\mu _{FL}}$(Fig. 4A, C, and E) and better scaffolds only if *C_R_* was >1× (Fig. 4D). Removal of variation had little effect on contigs and only gave rise to longer scaffolds if *C_R_* was >0.8× (Fig. S11), which is difficult to achieve with real libraries. Finally, a 1% uniform sequencing error had no discernible effect (Fig. S12).

### Performance of diploid assembly: fraction of genome in diploid state

While contiguity is an important parameter for any whole-genome assembly, evaluation of diploid assemblies necessitates estimating the fraction of the genome in which the assembly recovered the diploid state. To this end, we divided the contigs generated by Supernova2 into “diploid contigs,” which were extracted from its megabubble structures, and “haploid contigs” from non-megabubble structures. Pairs of scaffolds were extracted as the 2 haplotypes from megabubble structures if they shared the same start and end nodes in the assembly graph. Diploid contigs were generated by breaking the candidate scaffolds at the sequences with ≥10 consecutive Ns and were aligned to human reference genome (hg38) by Minimap2. The genome was split into 500-bp windows, and diploid regions were defined as the maximum extent of successive windows covered by 2 contigs, each from 1 haplotype. Alignment against the human reference genome revealed the overall genome coverages of the 6 assemblies to be ∼91%. For most assemblies, 70%–80% of the genome was covered by 2 homologous contigs (Table 1), with ${R_6}$ only reaching 58.9%, probably owing to the short fragments of the DNA preparation (${\mu _{FL}}$= 24 kb). We also analyzed another 7 assemblies produced by 10x Genomics, all of which had diploid fractions of ∼80% as well (Table S5). In the male NA24385, non-pseudoautosomal regions of the X chromosome are hemizygous and should therefore be recovered as haploid regions. Between 79.9% and 87.6% of these regions were covered by 1 contig exactly depending on the assembled library. Library construction parameters other than fragment length seemed to have had little effect on the proportion of diploid regions (Table 1 and Table S5).

**Table 1:** Genomic coverage of contigs generated by Supernova2

Linked-Reads set	Overall (%)	Diploid regions (%)	Haploid regions (%)	Non-pseudoautosomal regions of X chromosome (%)	Total contig length (contig > 500 bp)	Length of contigs from megabubble (contig > 500 bp)	Percentage (%)
${R_6}$	91.9	58.9	27.7		5,632,483,053	3,758,345,846	66.73
${R_7}$	91.1	73.3	11.3		5,613,140,437	4,668,186,478	83.17
${R_8}$	91.7	77.2	9.2		5,635,127,471	4,896,821,850	86.90
${R_9}$	91.3	73.4	12.2	85.9	5,637,615,919	4,438,175,621	78.72
${R_{10}}$	91.7	79.2	5.8	79.9	5,749,001,471	4,793,226,150	83.37
${R_{11}}$	91.7	78.1	7.9	87.6	5,677,566,094	4,723,083,367	83.19

*R*_6_, *R*_7_, and *R*_8_ are female; *R*_9_, *R*_10_, and *R*_11_ are male.

Overlapping the diploid regions from the assemblies of the same individual revealed that 50.24% and 67.27% of the genome for NA12878 and NA24385 (Fig. S13), respectively, were diploid in all 3 assemblies. NA12878 was lower because of the low percentage of diploid regions in assembly ${R_6}$ (Table 1). The overlaps were significantly greater than expected by chance (NA12878: 33.3%, *P*-value = 0.0049; NA24385: 45.4%, *P*-value = 0.0029, χ^2^ test). These observations were consistent with heterozygous variants being enriched in certain genomic segments, in which 2 haplotypes were more easily differentiated by Supernova2. Phase block lengths were mainly determined by total coverage *C* and increased in real data with increasing fragment length (Fig. S14, Table S6).

### Performance of diploid assembly: quality of variant calls

The ultimate goal of human genome assembly is to accurately identify genomic variants. We therefore compared the SNVs and SVs from our assemblies with the calls from referenced-based processing of standard Illumina and 10x data, and benchmarked them using gold standard from GIAB [42, 46] and PacBio CCS reads [44]. The accuracy of SNV calls from reference-based processing of standard Illumina and 10x data was comparable, but both were better than assembly-based calls (Tables S7 and S8). The likely reason for the relatively poor performance of assembly-based SNV calls is that the assemblies contain only ∼80% of the genome in a diploid state. For SVs, our assemblies generated many calls that were missed by the reference-based strategy (Tables S9–S12) and even by the Tier 1 benchmark of GIAB (Table S13), and half of the deletions and a majority of insertions could be validated by PacBio CCS reads (Table S14).

## Discussion

In the present study, we investigated human diploid assembly using 10x Linked-Read sequencing data on both simulated and real libraries. We developed the simulator LRTK-SIM to examine the likely impact of parameters in diploid assembly and compared results from simulated reads with those from real libraries. We thus determined the impact of key parameters (*C_R_, C_F_, N_F/P_*, and ${\mu _{FL}}$/*W*${\mu _{FL}}$) with respect to assembly continuity and accuracy. Our study provides a general strategy to evaluate assemblies of 10x data and may have implications for the evaluation of other barcode-based sequencing technologies such as CPTv2-seq [47] or stLRF [48] in the future.

### 10x Practicalities

For standard Illumina sequencing, library complexity is usually sufficient to generate tremendous numbers of reads from unique templates and read coverage can be increased simply by sequencing more. However, the 10x Chromium system performs amplification in each partition, and generally only ∼20–40% of the original long fragment sequence can be captured as short fragments and eventually as reads, resulting in shallow sequencing coverage per fragment. Sequencing more deeply does not increase the per-fragment coverage much because most of the extra reads are from PCR duplicates. The solution is to sequence multiple 10x libraries constructed from the same DNA preparation and merge them for analysis. This means that *C_R_* remains in the standard range where PCR duplicates are relatively rare, but *C_F_* increases proportionally to the number of libraries used. A practical limitation to this approach is that Supernova2 limits the number of barcodes to 4.8 million.

Our results showed that in practice, *C_F_* should be between 335× and 823×, but no larger than 1,000×, given the optimal coverage of *C* = 56× recommended by 10x and the requirement for sufficient per-fragment read coverage. Surprisingly, we observed that including more extremely long fragments was detrimental for assembly quality. This is possibly due to the loss of barcode specificity for fragments spanning repetitive sequences. From a computational perspective, too many long fragments are harmful to deconvolving the de Bruijn graph because more complex paths need to be picked out. In our experiments, *W*${\mu _{FL}}$ between 50 and 150 kb is the best choice to generate reliable assemblies.

### Parameters driving assembly quality

Our results regarding assembly quality, and the 10x parameters that influence it, may be useful for efforts in which *de novo* assemblies are important for generation of an initial reference sequence. We show that maximization of N50 does not necessarily reflect assembly quality, which we were able to compare to NA50 because there exists a high-quality human reference genome. Contig and scaffold lengths mostly increased with ascending sequencing coverage, and at sufficient overall sequence coverage it did not matter much whether the increasing coverage *C* was accomplished by increasing *C_R_* or *C_F_*. However, both contig and scaffold accuracy decreased with increasing *C*. We also found, counterintuitively, that contig and scaffold length mostly decreased with increasing fragment length, a phenomenon that may be due to the specific implementation; however, until there is another assembler that can be compared to Supernova2 it will not be possible to reason about this effect. In addition, intrinsic properties of the genome matter greatly because removal of repeats or lack of variation dramatically improves assembly quality.

Diploid assembly is the appropriate approach for assembly of genomes of diploid organisms that harbor variation. Therefore, an important metric to evaluate diploid assembly is the fraction of the genome that is assembled in a diploid state. The short input fragment length of ${R_6}$ resulted in ∼20% less of the genome in a diploid state (<60% vs <80%) compared to the other libraries of the same individual. This observation suggests that in addition to metrics such as N50, evaluation of assembly quality should also include the fraction of the genome (or the assembly) that is in a diploid state.

### Cost-benefit analysis

Overall, we have attempted to give practical guidelines to assembly of 10x data with Supernova2 and evaluate the performance across a wide range of metrics. Arguably, the metric that matters most in the context of a personal genome is the discovery of variation that lower-cost approaches do not enable. We estimate that the cost increase over standard Illumina sequencing is ∼2×, given the 10x preparation cost and the higher level of sequence coverage required. There may be many applications for which this combination of excellent SNV detection (via barcode-aware read mapping) and precise SV discovery (via assembly), achieved by the same data set, is worth the price.

### Comparison with hybrid assemblies

Hybrid assembly strategies have been applied successfully to produce human genome assembly of long contiguity [13, 14, 49]. In these studies, long contigs are first produced by single-molecule long reads, such as PacBio (NG50 = 1.1Mb [13]) or Nanopore (NG50 = 3.21 Mb [14]), comparing favorably to our best results for Linked-Reads assemblies (NG50 = 236 kb). Scaffolding is then performed with complementary technologies such as BioNano to capture chromosome-level long-range information. It promoted the scaffold N50 of PacBio to 31.1 Mb [13] and Illumina mate-pair sequencing with 10x data to 33.5 Mb [25]. Using SuperNova2, the scaffold N50 from our studies reached ∼27.86 Mb (${R_6}$) on the basis of 10x data alone, suggesting that 10x technology gives broadly comparable results at a fraction of the price of long-read–based hybrid assemblies.

## Availability of Supporting Data and Materials

The raw sequencing data are deposited in the Sequence Read Archive, and the corresponding BioProject accession number is PRJNA527321. Diploid assemblies and the codes for comparison are currently available at http://mendel.stanford.edu/supplementarydata/zhang_SN2_2019 and https://github.com/zhanglu295/Evaluate_diploid_assembly. LRTK-SIM is publicly available at https://github.com/zhanglu295/LRTK-SIM. Additional supporting data are available in the *GigaScience* GigaDB database [50].

## Additional Files

**Table S1**. Parameters of libraries prepared for NA12878 and NA24385.

**Table S2**. Parameters used to generate Linked-Read sets for evaluating the impact of *C_F_* and *C_R_* on assemblies.

**Table S3**. Parameters used to generate Linked-Read sets for evaluating the impact of ${\mu _{FL}}$ and *N_F/P_* on assemblies.

**Table S4**. Contig misassemblies and recovered transcripts of the 6 assemblies.

**Table S5**. Genomic coverage and fraction of contigs in diploid state generated by Supernova2 for the 7 libraries prepared by 10x Genomics. Non-PAR: non-pseudoautosomal regions of X chromosome. WFU, YOR, YORM, and PR are female; HGP, ASH, and CHI are male.

**Table S6**. Phase block N50s of the 6 assemblies.

**Table S7**. Comparison SNV calls from standard Illumina data, 10x reference-based calls, and assembly-based calls for NA12878. All calls were compared to the GIAB benchmark.

**Table S8**. Comparison SNV calls from standard Illumina data, 10x reference-based calls, and assembly-based calls for NA24385. All calls were compared to the GIAB benchmark.

**Table S9**. Comparison of SV calls from standard Illumina data and 10x assembly-based calls for NA12878.

**Table S10**. Comparison of SV calls from standard Illumina data and 10x assembly-based calls for NA24385.

**Table S11**. Comparison of SV calls from 10x reference-based and assembly-based calls for NA12878.

**Table S12**. Comparison of SV calls from 10x reference-based and assembly-based calls for NA24385.

**Table S13**. Comparison of SV calls from our *de novo* assemblies with the Tier 1 SV benchmark from GIAB.

**Table S14**. Proportion of assembly-based SV calls supported by PacBio CCS reads.

**Figure S1**. Basic statistics for ${L_{1L}}$. The distributions of **A**. the number of fragments per partition; **B**. sequencing depth per fragment; **C**. probability density function of unweighted fragment lengths; **D**. cumulative density function of unweighted fragment lengths; **E**. reversed cumulative density function of unweighted fragment lengths; **F**. reversed cumulative density function of weighted fragment lengths.

**Figure S2**. Basic statistics for ${L_{1M}}$. The distributions of **A**. number of fragments per partition; **B**. sequencing depth per fragment; **C**. probability density function of unweighted fragment lengths; **D**. cumulative density function of unweighted fragment lengths; **E**. reversed cumulative density function of unweighted fragment lengths; **F**. reversed cumulative density function of weighted fragment lengths.

**Figure S3**. Basic statistics for ${L_{1H}}$. The distributions of **A**. number of fragments per partition; **B**. sequencing depth per fragment; **C**. probability density function of unweighted fragment lengths; **D**. cumulative density function of unweighted fragment lengths; **E**. reversed cumulative density function of unweighted fragment lengths; **F**. reversed cumulative density function of weighted fragment lengths.

**Figure S4**. Basic statistics for ${L_2}$. The distributions of **A**. number of fragments per partition; **B**. sequencing depth per fragment; **C**. probability density function of unweighted fragment lengths; **D**. cumulative density function of unweighted fragment lengths; **E**. reversed cumulative density function of unweighted fragment lengths; **F**. reversed cumulative density function of weighted fragment lengths.

**Figure S5**. Basic statistics for ${L_3}$. The distributions of **A**. number of fragments per partition; **B**. sequencing depth per fragment; **C**. probability density function of unweighted fragment lengths; **D**. cumulative density function of unweighted fragment lengths; **E**. reversed cumulative density function of unweighted fragment lengths; **F**. reversed cumulative density function of weighted fragment lengths.

**Figure S6**. Basic statistics for ${L_4}$. The distributions of **A**. number of fragments per partition; **B**. sequencing depth per fragment; **C**. probability density function of unweighted fragment lengths; **D**. cumulative density function of unweighted fragment lengths; **E**. reversed cumulative density function of unweighted fragment lengths; **F**. reversed cumulative density function of weighted fragment lengths.

**Figure S7**. Basic statistics for ${L_5}$. The distributions of **A**. number of fragments per partition; **B**. sequencing depth per fragment; **C**. probability density function of unweighted fragment lengths; **D**. cumulative density function of unweighted fragment lengths; **E**. reversed cumulative density function of unweighted fragment lengths; **F**. reversed cumulative density function of weighted fragment lengths.

**Figure S8**. Basic statistics for ${L_6}$. The distributions of **A**. number of fragments per partition; **B**. sequencing depth per fragment; **C**. probability density function of unweighted fragment lengths; **D**. cumulative density function of unweighted fragment lengths; **E**. reversed cumulative density function of unweighted fragment lengths; **F**. reversed cumulative density function of weighted fragment lengths.

**Figure S9**. The workflow of LRTK-SIM to simulate Linked-Reads.

**Figure S10**. The effect of *N_F/P_* on human diploid assembly of chromosome 19 by Supernova2, where *C* (*C* = 60×; *C_F_* = 300× and *C_R_* = 0.2×) and ${\mu _{FL}}$ (${\mu _{FL}}$= 37 kb) are fixed.

**Figure S11**. Comparison of assembly qualities from 10x data with and without SNVs by changing *C_F_, C_R_*, and ${\mu _{FL}}$. *C_R_* was fixed to 0.2× in **A** and **B**; *C_F_* was fixed to 300× in **C** and **D**; *C_R_* was fixed to 0.2× and *C_F_* was fixed to 300× in **E** and **F**.

**Figure S12**. Comparison of assembly qualities from 10x data with (1% uniform) and without sequencing error by changing *C_F_, C_R_*, and ${\mu _{FL}}$. *C_R_* was fixed to 0.2× in **A** and **B**; *C_F_* was fixed to 300× in **C** and **D**; *C_R_* was fixed to 0.2× and *C_F_* was fixed to 300× in **E** and **F**.

**Figure S13**. Overlaps of diploid regions for the 3 libraries from the same sample. Diploid regions for NA12878 (**A**) and NA24385 (**B**). The percentages denote the proportion of genome that is diploid.

**Figure S14**. Phase block N50s as a function of different parameter combinations. **A**. Simulated Linked-Reads with predefined parameters (Table S5) by changing *C_F_* and *C_R_*; **B**. simulated Linked-Reads with matched parameters of real Linked-Read sets (Table S2) by changing *C_F_* and *C_R_*; **C**. real Linked-Read sets (Table S2) by changing *C_F_* and *C_R_*; **D**. simulated Linked-Read sets (Table S3) with different $W{\mu _{FL}}$; **E**. simulated Linked-Read sets with matched parameters (Table S3) with real Linked-Read sets as *C* = 56×; **F**. real Linked-Read sets with *C* = 56× (Table S3).

giz141_GIGA-D-19-00092_Original_Submission

giz141_GIGA-D-19-00092_Revision_1

giz141_GIGA-D-19-00092_Revision_2

giz141_Response_to_Reviewer_Comments_Original_Submission

giz141_Response_to_Reviewer_Comments_Revision_1

giz141_Reviewer_1_Report_Original_Submission Brock Peters, Ph.D. -- 5/23/2019 Reviewed

giz141_Reviewer_2_Report_Original_Submission Tobias Marschall -- 5/29/2019 Reviewed

giz141_Reviewer_2_Report_Revision_1 Tobias Marschall -- 10/17/2019 Reviewed

giz141_Supplemental_File

## Abbreviations

bp: base pairs; BWA: Burrows-Wheeler Aligner; CCS: Circular Consensus Sequencing; GIAB: Genome in a Bottle; HLA: human leukocyte antigen; kb: kilobase pairs; LFR: long fragment read; Mb: megabase pairs; ONT: Oxford Nanopore; PacBio: Pacific Biosciences; SNV: single-nucleotide variant; SV: structural variant.

## Competing Interests

A.S. is a consultant and shareholder of DNAnexus, Inc.

## Authors’ contributions

A.S. conceived the study. L.Z. and X.Z. wrote LRTK-SIM and performed the analyses. Z.W. prepared the genomic DNA and 10x libraries. L.Z., X.Z., Z.W., and A.S. analyzed the results and wrote the paper. All authors read and approved the final manuscript.

## References

[1] Metzker ML Sequencing technologies - the next generation. Nat Rev Genet. 2010;11(1):31–46. 19997069 10.1038/nrg2626

[2] Shendure J, Balasubramanian S, Church GM, et al. DNA sequencing at 40: past, present and future. Nature. 2017;550(7676):345–53. 29019985 10.1038/nature24286

[3] Head SR, Komori HK, LaMere SA, et al. Library construction for next-generation sequencing: overviews and challenges. BioTechniques. 2014;56(2):61–4., 6, 8, passim. 24502796 10.2144/000114133 PMC4351865

[4] O'Connell J, Sharp K, Shrine N, et al. Haplotype estimation for biobank-scale data sets. Nat Genet. 2016;48(7):817–20. 27270105 10.1038/ng.3583 PMC4926957

[5] Delaneau O, Zagury JF, Marchini J Improved whole-chromosome phasing for disease and population genetic studies. Nat Methods. 2013;10(1):5–6. 23269371 10.1038/nmeth.2307

[6] O'Connell J, Gurdasani D, Delaneau O, et al. A general approach for haplotype phasing across the full spectrum of relatedness. PLoS Genet. 2014;10(4):e1004234. 24743097 10.1371/journal.pgen.1004234 PMC3990520

[7] Roach JC, Glusman G, Hubley R, et al. Chromosomal haplotypes by genetic phasing of human families. Am J Hum Genet. 2011;89(3):382–97. 21855840 10.1016/j.ajhg.2011.07.023 PMC3169815

[8] Kajitani R, Toshimoto K, Noguchi H, et al. Efficient de novo assembly of highly heterozygous genomes from whole-genome shotgun short reads. Genome Res. 2014;24(8):1384–95. 24755901 10.1101/gr.170720.113 PMC4120091

[9] Alkan C, Sajjadian S, Eichler EE Limitations of next-generation genome sequence assembly. Nat Methods. 2011;8(1):61–5. 21102452 10.1038/nmeth.1527 PMC3115693

[10] Treangen TJ, Salzberg SL Repetitive DNA and next-generation sequencing: computational challenges and solutions. Nat Rev Genet. 2011;13(1):36–46. 22124482 10.1038/nrg3117 PMC3324860

[11] Huddleston J, Ranade S, Malig M, et al. Reconstructing complex regions of genomes using long-read sequencing technology. Genome Res. 2014;24(4):688–96. 24418700 10.1101/gr.168450.113 PMC3975067

[12] Lu H, Giordano F, Ning Z Oxford nanopore MinION sequencing and genome assembly. Genomics Proteomics Bioinformatics. 2016;14(5):265–79. 27646134 10.1016/j.gpb.2016.05.004 PMC5093776

[13] Pendleton M, Sebra R, Pang AW, et al. Assembly and diploid architecture of an individual human genome via single-molecule technologies. Nat Methods. 2015;12(8):780–6. 26121404 10.1038/nmeth.3454 PMC4646949

[14] Jain M, Koren S, Miga KH, et al. Nanopore sequencing and assembly of a human genome with ultra-long reads. Nat Biotechnol. 2018;36(4):338–45. 29431738 10.1038/nbt.4060 PMC5889714

[15] Wenger AM, Peluso P, Rowell WJ, et al. Accurate circular consensus long-read sequencing improves variant detection and assembly of a human genome. Nat Biotechnol. 2019;37(10):1155–62. 31406327 10.1038/s41587-019-0217-9 PMC6776680

[16] Cao H, Wu H, Luo R, et al. De novo assembly of a haplotype-resolved human genome. Nat Biotechnol. 2015;33(6):617–22. 26006006 10.1038/nbt.3200

[17] Kuleshov V, Xie D, Chen R, et al. Whole-genome haplotyping using long reads and statistical methods. Nat Biotechnol. 2014;32(3):261–6. 24561555 10.1038/nbt.2833 PMC4073643

[18] Peters BA, Kermani BG, Sparks AB, et al. Accurate whole-genome sequencing and haplotyping from 10 to 20 human cells. Nature. 2012;487(7406):190–5. 22785314 10.1038/nature11236 PMC3397394

[19] Edge P, Bafna V, Bansal V HapCUT2: robust and accurate haplotype assembly for diverse sequencing technologies. Genome Res. 2017;27(5):801–12. 27940952 10.1101/gr.213462.116 PMC5411775

[20] Patterson M, Marschall T, Pisanti N, et al. WhatsHap: weighted haplotype assembly for future-generation sequencing reads. J Comput Biol. 2015;22(6):498–509. 25658651 10.1089/cmb.2014.0157

[21] Zheng GX, Lau BT, Schnall-Levin M, et al. Haplotyping germline and cancer genomes with high-throughput linked-read sequencing. Nat Biotechnol. 2016;34(3):303–11. 26829319 10.1038/nbt.3432 PMC4786454

[22] Spies N, Weng Z, Bishara A, et al. Genome-wide reconstruction of complex structural variants using read clouds. Nat Methods. 2017;14(9):915–20. 28714986 10.1038/nmeth.4366 PMC5578891

[23] Bishara A, Moss EL, Kolmogorov M, et al. High-quality genome sequences of uncultured microbes by assembly of read clouds. Nat Biotechnol. 2018;36:1067–75. 10.1038/nbt.4266 PMC6465186 30320765

[24] Weisenfeld NI, Kumar V, Shah P, et al. Direct determination of diploid genome sequences. Genome Res. 2017;27(5):757–67. 28381613 10.1101/gr.214874.116 PMC5411770

[25] Mostovoy Y, Levy-Sakin M, Lam J, et al. A hybrid approach for de novo human genome sequence assembly and phasing. Nat Methods. 2016;13(7):587–90. 27159086 10.1038/nmeth.3865 PMC4927370

[26] Hulse-Kemp AM, Maheshwari S, Stoffel K, et al. Reference quality assembly of the 3.5-Gb genome of *Capsicum annuum* from a single linked-read library. Hortic Res. 2018;5:4. 29423234 10.1038/s41438-017-0011-0 PMC5798813

[27] Elyanow R, Wu HT, Raphael BJ Identifying structural variants using linked-read sequencing data. Bioinformatics. 2017;34(2):353–60. 10.1093/bioinformatics/btx712 PMC5860216 29112732

[28] Jones SJ, Haulena M, Taylor GA, et al. The genome of the northern sea otter (*Enhydra lutris kenyoni*). Genes (Basel). 2017;8(12):379. 10.3390/genes8120379 PMC5748697 29232880

[29] Genome In A Bottle Data ftp://ftp-trace.ncbi.nlm.nih.gov/giab/ftp/release/NA12878_HG001/latest/GRCh38/HG001_GRCh38_GIAB_highconf_CG-IllFB-IllGATKHC-Ion-10X-SOLID_CHROM1-X_v.3.3.2_highconf_nosomaticdel_noCENorHET7.bed. Accessed Nov 2019.

[30] Luo R, Sedlazeck FJ, Darby CA, et al. LRSim: A linked-reads simulator generating insights for better genome partitioning. Comput Struct Biotechnol J. 2017;15:478–84. 29213995 10.1016/j.csbj.2017.10.002 PMC5711661

[31] Li H, Handsaker B, Wysoker A, et al. The Sequence Alignment/Map format and SAMtools. Bioinformatics. 2009;25(16):2078–9. 19505943 10.1093/bioinformatics/btp352 PMC2723002

[32] Li H Minimap2: pairwise alignment for nucleotide sequences. Bioinformatics. 2018;34(18):3094–100. 29750242 10.1093/bioinformatics/bty191 PMC6137996

[33] Mikheenko A, Prjibelski A, Saveliev V, et al. Versatile genome assembly evaluation with QUAST-LG. Bioinformatics. 2018;34(13):i142–i50. 29949969 10.1093/bioinformatics/bty266 PMC6022658

[34] Earl D, Bradnam K, St John J, et al. Assemblathon 1: a competitive assessment of de novo short read assembly methods. Genome Res. 2011;21(12):2224–41. 21926179 10.1101/gr.126599.111 PMC3227110

[35] Zook JM, Catoe D, McDaniel J, et al. Extensive sequencing of seven human genomes to characterize benchmark reference materials. Sci Data. 2016;3:160025. 27271295 10.1038/sdata.2016.25 PMC4896128

[36] eWala JA, Bandopadhayay P, Greenwald NF, et al. SvABA: genome-wide detection of structural variants and indels by local assembly. Genome Res. 2018;28(4):581–91. 29535149 10.1101/gr.221028.117 PMC5880247

[37] Li H, Durbin R Fast and accurate short read alignment with Burrows-Wheeler transform. Bioinformatics. 2009;25(14):1754–60. 19451168 10.1093/bioinformatics/btp324 PMC2705234

[38] Garrison E, Marth G Haplotype-based variant detection from short-read sequencing. arXiv e-prints. 2012:1207.3907.

[39] What is long ranger? https://support.10xgenomics.com/genome-exome/software/pipelines/latest/what-is-long-ranger. Accessed Nov 2019

[40] NA12878 ftp://ftp-trace.ncbi.nlm.nih.gov/giab/ftp/release/NA12878_HG001/latest/GRCh38/. Accessed Nov 2019.

[41] NA24385 ftp://ftp-trace.ncbi.nlm.nih.gov/giab/ftp/release/AshkenazimTrio/HG002_NA24385_son/latest/GRCh38/. Accessed Nov 2019.

[42] Zook JM, Hansen NF, Olson ND, et al. A robust benchmark for germline structural variant detection. bioRxiv. 2019, doi:10.1101/664623.

[43] Zhao X, Weber AM, Mills RE A recurrence-based approach for validating structural variation using long-read sequencing technology. Gigascience. 2017;6(8):1–9. 10.1093/gigascience/gix061 PMC5737365 28873962

[44] Wenger AM, Peluso P, Rowell WJ, et al. Accurate circular consensus long-read sequencing improves variant detection and assembly of a human genome. Nat Biotechnol. 2019;37(10):1155–62. 31406327 10.1038/s41587-019-0217-9 PMC6776680

[45] Krusche P, Trigg L, Boutros PC, et al. Best practices for benchmarking germline small-variant calls in human genomes. Nat Biotechnol. 2019;37(5):555–60. 30858580 10.1038/s41587-019-0054-x PMC6699627

[46] Zook JM, McDaniel J, Olson ND, et al. An open resource for accurately benchmarking small variant and reference calls. Nat Biotechnol. 2019;37(5):561–6. 30936564 10.1038/s41587-019-0074-6 PMC6500473

[47] Zhang F, Christiansen L, Thomas J, et al. Haplotype phasing of whole human genomes using bead-based barcode partitioning in a single tube. Nat Biotechnol. 2017;35(9):852–7. 28650462 10.1038/nbt.3897

[48] Wang O, Chin R, Cheng X, et al. Efficient and unique cobarcoding of second-generation sequencing reads from long DNA molecules enabling cost-effective and accurate sequencing, haplotyping, and de novo assembly. Genome Res. 2019;29(5):798–808. 30940689 10.1101/gr.245126.118 PMC6499310

[49] Ma ZS, Li L, Ye C, et al. Hybrid assembly of ultra-long Nanopore reads augmented with 10x-Genomics contigs: demonstrated with a human genome. Genomics. 2018;111(6):1896–901. 30594583 10.1016/j.ygeno.2018.12.013

[50] Zhang L, Zhou X, Weng Z, et al. Supporting data for “Assessment of human diploid genome assembly with 10x Linked-Reads data.”. GigaScience Database. 2019 10.5524/100668. PMC6879002 31769805

