# Supplementary material for: Assessment of human diploid genome assembly with 10x Linked-Reads data
Source: Gigascience. 2019 Nov 26;8(11):giz141. doi: 10.1093/gigascience/giz141 (PMC6879002; doi:10.1093/gigascience/giz141)
Supplement: giz141_Response_to_Reviewer_Comments_Original_Submission [file giz141_response_to_reviewer_comments_original_submission.pdf]

# Author's Response To Reviewer Comments

Close

Reviewer reports:

Reviewer #1: Zhang et al. explore the parameter space of 10X libraries and the subsequent effects of those parameters on de novo assembly performance. They also developed an in silico simulator and that generates results similar to experimental findings. The manuscript is well written and easy to understand.

We thank the reviewer for these positive comments and address each point below.

That said, I think there are some analyses missing that should be included:

1. I think you should variant call off of the de novo assemblies to see if there are any differences you are missing because you're only looking at things at a very high structural level.

We have now called SNVs and SVs from our de novo assemblies and from other methods. Please find our results in the responses to points 2-4 of reviewer2.

2. How is phasing affected? I don't see any data on that other than total diploid regions. You should include the changes to the phase block N50. It's mentioned in the abstract, but I don't see it anywhere else.

We have showed the trend of phased block N50 in different linked-read sets in Figure S14, now we also provided the values of phase block N50s in Table S6

3. Besides NA50 you should include assembly errors such as breakpoints, translocations, inversions, relocations, etc…..
You have a nice dataset here, you should try to get more out of it.

Thank you for the suggestions. We have re-run QUAST and generated several detailed statistics which are now shown in Table S4. These results are consistent with the contig N50s reported in Figure 3.

Minor comments:
58-66, Probably should add this reference for PacBio CCS sequencing, contig N50 is 15 mb, https://www.biorxiv.org/content/10.1101/519025v2
We have added this reference

65-66, I'd argue that this statement is a bit strong, cost is lowering, and throughput is increasing for these systems
This is now lines 70-72. We have rephrased the sentence and now write: "However, long-fragment sequencing suffers from extremely high cost (in the case of PacBio CCS), or low base quality (in the case of single-pass reads of either technology), hampering its usefulness for personal genome assembly."

68 Not a complete sentence
We fixed this

Ref 27 isn't our stLFR paper, the doi for that is 10.1101/gr.245126.118, and it is commercially available now in some parts of the world
We have added the new reference and deleted the confusing words in this sentence.

Reviewer #2: Zhang and co-authors present a parameter study for 10x linked-read sequencing experiments with the objective of evaluating the influence of experimentally controllable parameters on the final diploid assembly quality. The authors perform basic performance evaluation in terms of common metrics such as N50 values and provide technical recommendations for designing linked-read

sequencing experiments. Additionally, Zhang et al. implemented a software tool for simulating linked-read sequencing data, which they use for parameter assessment given the known (simulated) truth.

While such studies that provide guidance to users of a sequencing technology are very valuable in principle, I have a number of concerns that should be addressed:

1. There is a closely related article by Luo et al. (2017, DOI: 10.1016/j.csbj.2017.10.002) that has been missed. The authors should clarify what the added value of their study is beyond the work by Luo et al. This comment applies to both aspects: guidance to users in terms of 10x sequencing experiments and the utility/features of their data simulation tool (note that Luo et al. also provide a simulator).

We appreciate and cite the work by Luo et al. However, our study provides (1) a more flexible simulation tool and (2) an extensive set of new sequence data.

Regarding (1)
A. We explicitly allow users to input CF, CR, Wμ_FL and μ_FL, which have strong connections with library preparation and Illumina sequencing. For example, CF is driven by input DNA amount and μ_FL by DNA preparation and potential size selection. LRSIM only lets the user set the total number of reads.
B. The usability of LRTK-SIM is better than LRSIM. LRSIM requires many third party packages and software to be installed first, such as Inline::C perl library, DWGSIM etc. It is not convenient for the users with insufficient computer experience. LRTK-SIM was written in Python and no third-party software was required. It can be installed and gotten started easily. LRTK-SIM can parallel simulate multiple libraries with a variety of parameters simultaneously. The users can compare the performance of different parameters in one run.

Regarding (2)
Luo et al. compared the influence of different parameters by simulation only, which does not always reflect the situation in real sequencing. In our study, we prepared six real libraries with different parameters and could validate our observations from simulation data.

2. The focus of this manuscript is on guiding researchers who are after a cost-effective characterization of individual human genomes. In my view, Zhang et al. should go the full distance and additionally compare to standard Illumina sequencing followed by mapping and variant calling as a baseline. The assembly metrics employed are not so very informative when it comes to the question of which variation (relative to the reference genome) is been missed/captured in standard approaches.

While human assembly is the focus, we believe that much of the interest in our work will come mainly from researchers who are interested in assembling novel genomes. We use human as an assembly model because assembly quality can be gauged by comparison to the reference sequence. Nonetheless ...

Beyond comparing to standard Illumina sequencing, including a detailed comparison to reference-based processing of 10x data (e.g. using LongRanger) would be interesting. In this way, this study would by much more helpful for planning sequencing studies.

... in response to this comment, we now systematically investigate SNV and SV calls from our assemblies. We compare with standard Illumina data and reference-based processing of our 10x data. The standard Illumina data were downloaded from Genome In A Bottle and analyzed with SVABA to generate SV calls, and with BWA and FreeBayes to generate SNV calls. Long ranger was used to generate SNVs and SVs (only deletions) for 10x reference-based analysis. We noted that R9 failed to be analyzed by Long Ranger due to its extremely large CF. We compared SNV and SV calls among the different approaches using vcfeval (https://github.com/RealTimeGenomics/rtg-tools) and truvari (https://github.com/spiralgenetics/truvari), respectively.

For SNVs, we compared the calls from three strategies to the gold standard of NA12878 (ftp://ftp-trace.ncbi.nlm.nih.gov/giab/ftp/release/NA12878_HG001/latest/GRCh38/) and NA24385 (ftp://ftp-trace.ncbi.nlm.nih.gov/giab/ftp/release/AshkenazimTrio/HG002_NA24385_son/latest/GRCh38/).

We found that SNVs from reference-based processing of Illumina and 10x data were comparable, and both of them were better than assembly-based SNV calls. For SVs, our assemblies generated many calls that were missed by the reference-based strategy.

We now provide several additional supplementary tables (Table S7-S12) to present these results.

3. The main reason (in my view) for pursuing de novo assembly of human genomes is to access structural variation that is missed otherwise. An evaluation on how much structural variation is (accurately) captured would be of interest to many readers. This is actually something that the authors point out in the Discussion themselves: "Arguably, the metric that matters most in the context of a personal genome is the discovery of variation that lower-cost approaches do not enable."

As implied by the quote, we agree with the reviewer's comment. Consequently, we now compare three linked-read sets from HG002 with the Tier 1 SV benchmark from Genome in a Bottle by using truvari (https://github.com/spiralgenetics/truvari). The results are summarized in Table S13.

4. PacBio CCS reads are available for HG002 (see Wenger at al., http://dx.doi.org/10.1101/519025). Mapping those CCS reads back to your diploid assemblies and calling variants provides an easy and powerful opportunity to assess the sequence quality from an independent technology.

These data became available while our manuscript was in review. We note that the PacBio CCS calls on HG002 are generally reasonably accurate but are not guaranteed to be correct in the absence of a gold standard. Therefore, we prefer to compare them in an overlap analysis with our calls, as opposed to implying that they are a gold standard by using the term "validation". We used vapor (https://github.com/mills-lab/vapor) to validate our SV calls based on PacBio CCS reads from HG002 and include Table S14 to show the validation rates.

Beyond this, your evaluation could be improved by also adding an assembly evaluation perspective that is more biologically motivated, e.g., number of recovered genes/disrupted genes or similar (this should be supported by Quast-LG/BUSCO).

We have added this analysis in Table S4.

Minor comments

- line 51: pedigree based phasing is quite powerful even for trios (where it is able to phase all variants that are homozygous in at least one individual), so I disagree to the statement that this is only feasible in large pedigrees.
We fixed this and removed confusing words.

- lines 60ff: it is unclear which study your are referring to here, please add the citation at the end of the sentence (N50 31.1Mb)
We included a new reference here.

- line 68: broken sentence; also, putting the citation at the end of the sentence increases readability
We fixed this issue.

- lines 71/72: again, unclear which study you are referring to ("Long Fragment Read")
We included a new reference here.

- lines 125ff: is there a specific reason why five and three? (And not, e.g., five and five?) Also, the meaning of L, M, and H in the subscript of L should be explained
Because we generated two additional libraries (L_1L and L_1M for NA12878) to evaluate the effects of CF and CR in assembly, and we believe the trend should be consistent in the two samples. L, M and H represent low, medium and high CF in the experiments. We have clarified this in the manuscript.

- line 129: percent of what?
The percent of GEM in 10x Chromium system.

- line 151: please be more specific about which version of hg38 was used (detail once if identical hg38 was used throughout the rest of the paper [lines 165, 171, 195 and so on...])
The reference was downloaded from 10x website with the version of GRCh38 Reference 2.1.0.

- line 172: please provide an exact reference for the high confidence regions that you used (e.g., file URL)
We have added the URL in the manuscript.

- line 208: "in in"
We fixed this.

- line 208: this sentence is talking about real data, so the reference to Fig 2C and 2D does not match.
We clarified this in the manuscript.

- line 209: "...but not dramatically... [...] ...appreciably" - this is subjective language, please rephrase and be more fact-oriented (for instance by including the numbers you refer to in parentheses).
We included the numbers and rephrased the sentence to be more fact-oriented.

- line 250: "_Alignment" ?
We fixed this.

- line 251: what is the denominator for these 91% all bases that are not Ns in the reference genome? (Note that for this analysis, the version of hg38 matters, see comment above).
"N"s do not contribute to the denominator.

- The authors mention stLFR in line 278. There's a new preprint that's worth citing/discussing: http://dx.doi.org/10.1101/324392
We have cited their latest version.

- line 296: "extremely long" please say what extremely long means here
We defined "extremely long" as the DNA fragments longer than 200kb.

- line 570: please be more specific what you mean by "in-house programs", and where the respective sources are available (is that the "Evaluate_diploid_assembly" github?)
All the source codes for assembly evaluation are available in https://github.com/zhanglu295/Evaluate_diploid_assembly. We added this information in the sentence.

- please add a - preferably open source - license file to your github repositories
We added the license files in the GitHub.

- "sample prep" is jargon and should be replaced by "sample preparation" (eg. line 41, but also elsewhere)
We have updated all the "sample prep" to "sample preparation" in the manuscripts.

Close
