# Supplementary material for: Assessment of human diploid genome assembly with 10x Linked-Reads data
Source: Gigascience. 2019 Nov 26;8(11):giz141. doi: 10.1093/gigascience/giz141 (PMC6879002; doi:10.1093/gigascience/giz141)
Supplement: giz141_Response_to_Reviewer_Comments_Revision_1 [file giz141_response_to_reviewer_comments_revision_1.pdf]

Author's Response To Reviewer Comments

Close

Reviewer reports:
Reviewer #2: The authors improved the manuscript substantially and implemented many of the suggested changes. I wonder, however, whether there was a mixup of document versions because not all changes described in the response are reflected in the manuscript (including trivial ones like fixing the "_Alignment", now in line 283; also Luo et al. is still not cited). Maybe the authors can double check that they indeed uploaded the latest version?

Thank you for pointing out this oversight. We have double-checked everything and added a section to the appropriate place in the Methods where we explain the differences between Luo et al's method and ours. (The added text is highlighted in red.)

Beyond that, the only concern left for me is the poor concordance of small variant calls. For the Illumina and 10x calls, my guess is that they went into the evaluation completely unfiltered, where FreeBayes (and the LongRanger pipeline which is based on FreeBayes) usually attain an acceptable precision only when the calls are filtered (e.g. for QUAL>=10). Much more concerning is the observation that between a quarter and half of all calls are missed by the assembly strategy. How did the authors call variants from the assemblies? Given that the GIAB benchmark regions are (comparatively) easy genomic regions, I think that the authors should offer an explanation for the poor recall.

We did not use any threshold to filter out low-quality variants from FreeBayes. To generate assembly-based calls, we aligned the two haploid contigs from Supernova to the reference genome (Mimimap2) independently and compared the two alleles of the corresponding coordinates (Paftools, mapQ>20).

For small SNV calls, we agree using Freebayes is a better choice since mapping-based algorithms have good base accuracy and assembly-based algorithms may lose sensitivity. The significant false negative rates of assembly-based calls likely come from two issues:
1. Supernova cannot guarantee to generate diploid contigs (megabubbles) even for the "easy regions" from GIAB, because the diploid contigs would be influenced by SV also. As a result, in those regions we lose a large fraction of heterozygous variants.
2. The single base variants in the de bruijn graph are represented as small bubbles, which would be flattened due to various reasons. The k-mer coverage is one of the critical thresholds, but the length of k-mer is much shorter than reads and the sequencing qualities are not taken into consideration. These may lead to miscount the coverage of variant alleles in the bubbles.

We have added an explanatory sentence in the last section of the Results.

Close
