# Supplementary material for: Assessment of human diploid genome assembly with 10x Linked-Reads data
Source: Gigascience. 2019 Nov 26;8(11):giz141. doi: 10.1093/gigascience/giz141 (PMC6879002; doi:10.1093/gigascience/giz141)
Supplement: giz141_Reviewer_1_Report_Original_Submission — Brock Peters, Ph.D. -- 5/23/2019 Reviewed [file giz141_reviewer_1_report_original_submission.pdf]

Reviewer Report

Title: Assessment of human diploid genome assembly with 10x Linked-Reads data

Version: Original Submission **Date:** 5/23/2019

Reviewer name: Brock Peters, Ph.D.

Reviewer Comments to Author:

Zhang et al. explore the parameter space of 10X libraries and the subsequent effects of those parameters on de novo assembly performance. They also developed an in silico simulator and that generates results similar to experimental findings. The manuscript is well written and easy to understand. That said, I think there are some analyses missing that should be included:

1. I think you should variant call off of the de novo assemblies to see if there are any differences you are missing because you're only looking at things at a very high structural level.
2. How is phasing affected? I don't see any data on that other than total diploid regions. You should include the changes to the phase block N50. It's mentioned in the abstract, but I don't see it anywhere else.
3. Besides NA50 you should include assembly errors such as breakpoints, translocations, inversions, relocations, etc…..

You have a nice dataset here, you should try to get more out of it.

Minor comments:

58-66, Probably should add this reference for PacBio CCS sequencing, contig N50 is 15 mb, https://www.biorxiv.org/content/10.1101/519025v2

65-66, I'd argue that this statement is a bit strong, cost is lowering, and throughput is increasing for these systems

68 Not a complete sentence

Ref 27 isn't our stLFR paper, the doi for that is 10.1101/gr.245126.118, and it is commercially available now in some parts of the world

Level of Interest

Please indicate how interesting you found the manuscript: Choose an item.

Quality of Written English

Please indicate the quality of language in the manuscript: Choose an item.

Declaration of Competing Interests

Please complete a declaration of competing interests, considering the following questions:

- Have you in the past five years received reimbursements, fees, funding, or salary from an organisation that may in any way gain or lose financially from the publication of this manuscript, either now or in the future?
- Do you hold any stocks or shares in an organisation that may in any way gain or lose financially from the publication of this manuscript, either now or in the future?
- Do you hold or are you currently applying for any patents relating to the content of the manuscript?
- Have you received reimbursements, fees, funding, or salary from an organization that holds or has applied for patents relating to the content of the manuscript?
- Do you have any other financial competing interests?
- Do you have any non-financial competing interests in relation to this paper?

If you can answer no to all of the above, write 'I declare that I have no competing interests' below. If your reply is yes to any, please give details below.

Yes, 5, I work for Complete Genomics/BGI, we develop DNA sequencing technology and I work on strategies similar to 10X Genomics technology. That said, this paper has little effect on what we are developing and if anything probably benefits our competitor 10X over us.

I agree to the open peer review policy of the journal. I understand that my name will be included on my report to the authors and, if the manuscript is accepted for publication, my named report including any attachments I upload will be posted on the website along with the authors' responses. I agree for my report to be made available under an Open Access Creative Commons CC-BY license (http://creativecommons.org/licenses/by/4.0/). I understand that any comments which I do not wish to be included in my named report can be included as confidential comments to the editors, which will not be published.

Choose an item.

To further support our reviewers, we have joined with Publons, where you can gain additional credit to further highlight your hard work (see: https://publons.com/journal/530/gigascience). On publication of this paper, your review will be automatically added to Publons, you can then choose whether or not to claim your Publons credit. I understand this statement.

Yes Choose an item.
