# Supplementary material for: Assessment of human diploid genome assembly with 10x Linked-Reads data
Source: Gigascience. 2019 Nov 26;8(11):giz141. doi: 10.1093/gigascience/giz141 (PMC6879002; doi:10.1093/gigascience/giz141)
Supplement: giz141_Reviewer_2_Report_Original_Submission — Tobias Marschall -- 5/29/2019 Reviewed [file giz141_reviewer_2_report_original_submission.pdf]

Reviewer Report

Title: Assessment of human diploid genome assembly with 10x Linked-Reads data

Version: Original Submission **Date:** 5/29/2019

Reviewer name: Tobias Marschall

Reviewer Comments to Author:

Zhang and co-authors present a parameter study for 10x linked-read sequencing experiments with the objective of evaluating the influence of experimentally controllable parameters on the final diploid assembly quality. The authors perform basic performance evaluation in terms of common metrics such as N50 values and provide technical recommendations for designing linked-read sequencing experiments. Additionally, Zhang et al. implemented a software tool for simulating linked-read sequencing data, which they use for parameter assessment given the known (simulated) truth.
While such studies that provide guidance to users of a sequencing technology are very valuable in principle, I have a number of concerns that should be addressed:
- There is a closely related article by Luo et al. (2017, DOI: 10.1016/j.csbj.2017.10.002) that has been missed. The authors should clarify what the added value of their study is beyond the work by Luo et al. This comment applies to both aspects: guidance to users in terms of 10x sequencing experiments and the utility/features of their data simulation tool (note that Luo et al. also provide a simulator).
- The focus of this manuscript is on guiding researchers who are after a cost-effective characterization of individual human genomes. In my view, Zhang et al. should go the full distance and additionally compare to standard Illumina sequencing followed by mapping and variant calling as a baseline. The assembly metrics employed are not so very informative when it comes to the question of which variation (relative to the reference genome) is been missed/captured in standard approaches. Beyond comparing to standard Illumina sequencing, including a detailed comparison to reference-based processing of 10x data (e.g. using LongRanger) would be interesting. In this way, this study would by much more helpful for planning sequencing studies.
- The main reason (in my view) for pursuing de novo assembly of human genomes is to access structural variation that is missed otherwise. An evaluation on how much structural variation is (accurately) captured would be of interest to many readers. This is actually something that the authors point out in the Discussion themselves: "Arguably, the metric that matters most in the context of a personal genome is the discovery of variation that lower-cost approaches do not enable."
- PacBio CCS reads are available for HG002 (see Wenger at al., http://dx.doi.org/10.1101/519025). Mapping those CCS reads back to your diploid assemblies and calling variants provides an easy and powerful opportunity to assess the sequence quality from an independent technology. Beyond this, your evaluation could be improved by also adding an assembly evaluation perspective that is more biologically motivated, e.g., number of recovered genes/disrupted genes or similar (this should be supported by Quast-LG/BUSCO).
Minor comments
- line 51: pedigree based phasing is quite powerful even for trios (where it is able to phase all variants

that are homozygous in at least one individual), so I disagree to the statement that this is only feasible in large pedigrees.
- lines 60ff: it is unclear which study your are referring to here, please add the citation at the end of the sentence (N50 31.1Mb)
- line 68: broken sentence; also, putting the citation at the end of the sentence increases readability
- lines 71/72: again, unclear which study you are referring to ("Long Fragment Read")
- lines 125ff: is there a specific reason why five and three? (And not, e.g., five and five?) Also, the meaning of L, M, and H in the subscript of L should be explained
- line 129: percent of what?
- line 151: please be more specific about which version of hg38 was used (detail once if identical hg38 was used throughout the rest of the paper [lines 165, 171, 195 and so on...])
- line 172: please provide an exact reference for the high confidence regions that you used (e.g., file URL)
- line 208: "in in"
- line 208: this sentence is talking about real data, so the reference to Fig 2C and 2D does not match.
- line 209: "...but not dramatically... [...] ...appreciably" - this is subjective language, please rephrase and be more fact-oriented (for instance by including the numbers you refer to in parentheses).
- line 250: "_Alignment" ?
- line 251: what is the denominator for these 91% all bases that are not Ns in the reference genome? (Note that for this analysis, the version of hg38 matters, see comment above).
- The authors mention stLFR in line 278. There's a new preprint that's worth citing/discussing: http://dx.doi.org/10.1101/324392
- line 296: "extremely long" please say what extremely long means here
- line 570: please be more specific what you mean by "in-house programs", and where the respective sources are available (is that the "Evaluate_diploid_assembly" github?)
- please add a - preferably open source - license file to your github repositories
- "sample prep" is jargon and should be replaced by "sample preparation" (eg. line 41, but also elsewhere)

Level of Interest

Please indicate how interesting you found the manuscript: Choose an item.

Quality of Written English

Please indicate the quality of language in the manuscript: Choose an item.

Declaration of Competing Interests

Please complete a declaration of competing interests, considering the following questions:

- Have you in the past five years received reimbursements, fees, funding, or salary from an organisation that may in any way gain or lose financially from the publication of this manuscript, either now or in the future?
- Do you hold any stocks or shares in an organisation that may in any way gain or lose financially from the publication of this manuscript, either now or in the future?
- Do you hold or are you currently applying for any patents relating to the content of the manuscript?
- Have you received reimbursements, fees, funding, or salary from an organization that holds or has applied for patents relating to the content of the manuscript?
- Do you have any other financial competing interests?
- Do you have any non-financial competing interests in relation to this paper?

If you can answer no to all of the above, write 'I declare that I have no competing interests' below. If your reply is yes to any, please give details below.

I declare that I have no competing interests

I agree to the open peer review policy of the journal. I understand that my name will be included on my report to the authors and, if the manuscript is accepted for publication, my named report including any attachments I upload will be posted on the website along with the authors' responses. I agree for my report to be made available under an Open Access Creative Commons CC-BY license (http://creativecommons.org/licenses/by/4.0/). I understand that any comments which I do not wish to be included in my named report can be included as confidential comments to the editors, which will not be published.

Choose an item.

To further support our reviewers, we have joined with Publons, where you can gain additional credit to further highlight your hard work (see: https://publons.com/journal/530/gigascience). On publication of this paper, your review will be automatically added to Publons, you can then choose whether or not to claim your Publons credit. I understand this statement.

Yes Choose an item.
