# Supplementary material for: Assessment of human diploid genome assembly with 10x Linked-Reads data
Source: Gigascience. 2019 Nov 26;8(11):giz141. doi: 10.1093/gigascience/giz141 (PMC6879002; doi:10.1093/gigascience/giz141)
Supplement: giz141_Reviewer_2_Report_Revision_1 — Tobias Marschall -- 10/17/2019 Reviewed [file giz141_reviewer_2_report_revision_1.pdf]

Reviewer Report

Title: Assessment of human diploid genome assembly with 10x Linked-Reads data

Version: Revision 1 **Date:** 10/17/2019

Reviewer name: Tobias Marschall

Reviewer Comments to Author:

The authors improved the manuscript substantially and implemented many of the suggested changes. I wonder, however, whether there was a mixup of document versions because not all changes described in the response are reflected in the manuscript (including trivial ones like fixing the "_Alignment", now in line 283; also Luo et al. is still not cited). Maybe the authors can double check that they indeed uploaded the latest version?
Beyond that, the only concern left for me is the poor concordance of small variant calls. For the Illumina and 10x calls, my guess is that they went into the evaluation completely unfiltered, where FreeBayes (and the LongRanger pipeline which is based on FreeBayes) usually attain an acceptable precision only when the calls are filtered (e.g. for QUAL>=10). Much more concerning is the observation that between a quarter and half of all calls are missed by the assembly strategy. How did the authors call variants from the assemblies? Given that the GIAB benchmark regions are (comparatively) easy genomic regions, I think that the authors should offer an explanation for the poor recall.

Level of Interest

Please indicate how interesting you found the manuscript: Choose an item.

Quality of Written English

Please indicate the quality of language in the manuscript: Choose an item.

Declaration of Competing Interests

Please complete a declaration of competing interests, considering the following questions:

- Have you in the past five years received reimbursements, fees, funding, or salary from an organisation that may in any way gain or lose financially from the publication of this manuscript, either now or in the future?
- Do you hold any stocks or shares in an organisation that may in any way gain or lose financially from the publication of this manuscript, either now or in the future?
- Do you hold or are you currently applying for any patents relating to the content of the manuscript?
- Have you received reimbursements, fees, funding, or salary from an organization that holds or has applied for patents relating to the content of the manuscript?

- Do you have any other financial competing interests?
- Do you have any non-financial competing interests in relation to this paper?

If you can answer no to all of the above, write 'I declare that I have no competing interests' below. If your reply is yes to any, please give details below.

I declare that I have no competing interests

I agree to the open peer review policy of the journal. I understand that my name will be included on my report to the authors and, if the manuscript is accepted for publication, my named report including any attachments I upload will be posted on the website along with the authors' responses. I agree for my report to be made available under an Open Access Creative Commons CC-BY license (http://creativecommons.org/licenses/by/4.0/). I understand that any comments which I do not wish to be included in my named report can be included as confidential comments to the editors, which will not be published.

Choose an item.

To further support our reviewers, we have joined with Publons, where you can gain additional credit to further highlight your hard work (see: https://publons.com/journal/530/gigascience). On publication of this paper, your review will be automatically added to Publons, you can then choose whether or not to claim your Publons credit. I understand this statement.

Yes Choose an item.
